# Editorial: Expanding the glial frontiers: development, function and pathophysiology

**DOI:** 10.3389/fncel.2026.1852203

**Published:** 2026-04-23

**Authors:** Dong Won Kim

**Affiliations:** 1 Danish Research Institute of Translational Neuroscience (DANDRITE), Nordic European Molecular Biology Laboratory, Partnership for Molecular Medicine, Aarhus University, Aarhus, Denmark; 2 Department of Biomedicine, Aarhus University, Aarhus, Denmark

**Keywords:** cell differentiation (glial development), energy metabolism (glial metabolism), myelination and remyelination, neuroglia, neuroglial cell plasticity

Glial biology has undergone a major conceptual shift over the past decades. Glial cells were previously viewed primarily as passive support elements, but are now recognized as active regulators of nervous system development, homeostasis, and disease. Across the central and peripheral nervous systems, glial populations regulate progenitor dynamics, metabolic support, synaptic and circuit function, myelin formation, tissue repair, and pathological responses. In parallel, increasing evidence demonstrates substantial heterogeneity within glial populations, shaped by developmental timing, anatomical location, and environmental context. Together, these advances move the field from a descriptive framework toward a systems-level view in which glial states actively influence neural function across the lifespan.

This Research Topic, *Expanding the glial frontiers: development, function and pathophysiology*, brings together six contributions that capture this expanded view. Despite differences in cell types, model systems, and experimental approaches, three common themes emerge. First, glial development is regulated through dynamic interactions with the microenvironment in addition to intrinsic programs. Second, the glial metabolic state is tightly coupled to both physiological function and disease processes. Third, myelin biology continues to provide a central framework linking glial specialization to structural and functional outcomes.

A first theme is the role of developmental context and cell-environment interactions. Al-Naama and Pearson examine radial glia at the neurovascular interface and show that progenitor-like radial glial cells both respond to and shape the developing vascular niche. Their analysis highlights coordinated signaling between neural progenitors and endothelial cells that couples angiogenesis, metabolic supply, and neurogenesis. In a complementary study, Sompub et al. investigate NF-κB RelA in oligodendrocyte-lineage cells and show that RelA regulates the temporal progression of oligodendrocyte differentiation. Rather than acting as a global determinant of lineage identity, it defines a temporally restricted differentiation program. Together, these studies support a model in which glial development is dynamically regulated by the interaction between transcriptional programs and local signaling environments.

A second theme is the functional role of glial metabolism. Ohguro et al. analyze Schwann cell responses under high-glucose conditions and show that imeglimin and metformin differentially regulate mitochondrial respiration, glycolysis, and reactive oxygen species production. These effects are further influenced by lipid-associated pathways, indicating that intracellular metabolic balance directly influences glial function in peripheral neuropathy. In a distinct context, Nasu and Terunuma review astrocyte contributions to depression and integrate evidence associating glutamate-glutamine cycling, lactate metabolism, mitochondrial function, ATP signaling, and gap-junction coupling to circuit dysfunction. Importantly, these studies support a model in which astrocytic dysfunction contributes directly to disease phenotypes rather than acting solely as a secondary response. Together, these findings position glial metabolism as a regulatory axis coupling cellular state to neural circuit activity and disease.

A third theme centers on myelin biology for understanding glial specialization and therapeutic intervention. Yamazaki and Ohno review clemastine-mediated remyelination and show that pharmacological enhancement of oligodendrocyte precursor differentiation can promote repair across multiple models. While these findings highlight therapeutic potential, they also underscore the complexity of remyelination mechanisms. Extending this perspective, Okuyama et al. survey multiscale imaging approaches to myelin, from histological to advanced *in vivo* imaging modalities. Their analysis emphasizes that myelin should be considered a dynamic and quantifiable substrate translating cellular processes to tissue-level function. Together, these studies reinforce the role of myelin biology as a bridge between development, pathology, and intervention.

Across these contributions, a unifying conceptual shift becomes evident. Glial biology is increasingly defined by regulated cellular states rather than static cell identities. Developmental stage, microenvironment, metabolic condition, and pathological stress collectively shape glial behavior. This perspective aligns with single-cell and functional studies showing that glial cells occupy dynamic and context-dependent states that influence neural systems.

Several key challenges remain. It will be necessary to define the regulatory programs governing glial state transitions, to determine how developmental and disease-associated states intersect, and to establish causal links between glial states and system-level phenotypes. Addressing these questions will require integrating single-cell profiling, spatial approaches, *in vivo* imaging, and perturbation-based experiments. Equally important will be the integration of findings across central and peripheral systems and across developmental and pathological contexts.

In summary, this Research Topic reflects the continued maturation of glial biology into a mechanistically grounded field. The studies presented here demonstrate that glial cells function as active regulators of nervous system organization and disease. We thank the contributing authors, reviewers, and the editorial team at *Frontiers in Cellular Neuroscience*. We hope this Research Topic will contribute to ongoing efforts to define how glial states shape neural function in both health and disease.

